# Advancing Precision Medicine in Degenerative Cervical Myelopathy

**DOI:** 10.3390/jcm14238344

**Published:** 2025-11-24

**Authors:** Abdul Al-Shawwa, David W. Cadotte

**Affiliations:** 1 Hotchkiss Brain Institute, Cumming School of Medicine, University of Calgary, Calgary, AB T2N 4N1, Canada; abduljawwad.alshawwa@ucalgary.ca; 2 Combined Orthopedic and Neurosurgery Spine Program, University of Calgary, Calgary, AB T2N 2T9, Canada; 3 Section of Neurosurgery, Department of Clinical Neurosciences, Cumming School of Medicine, University of Calgary, Calgary, AB T2N 2T9, Canada; 4 Department of Radiology, Cumming School of Medicine, University of Calgary, Calgary, AB T2N 2T9, Canada

**Keywords:** DCM, machine learning, cervical myelopathy, predictive model, prognostic, spinal cord injury, spinal cord compression, spinal canal stenosis, advanced imaging

## Abstract

Degenerative cervical myelopathy (DCM) is the leading cause of nontraumatic spinal cord dysfunction and remains clinically heterogeneous in presentation and course. This review synthesizes current evidence on predictors of neurological outcomes across conventional prognostic factors (clinical and macrostructural metrics) and quantitative neuroimaging (microstructural metrics), as well as how machine learning (ML) models integrate these predictors into a precision medicine framework to aid in DCM management. We explore evidence on conventional clinical and radiographic factors. Although several signs and scales are associated with clinical outcomes, cross-study inconsistency and the limits of linear models blunt their standalone utility, underscoring the need for multifactorial modelling. We then assess quantitative MRI biomarkers, including diffusion tensor imaging, magnetization transfer, and myelin water imaging, which index axonal integrity and myelination, thereby enriching risk stratification and prediction. Building on these measurements, we examine ML models combining clinical, imaging, and demographic features to predict postoperative outcomes and, increasingly, the natural history of mild DCM. Finally, current gaps and necessary future directions are outlined, including protocol harmonization, prospective multicentre validation, and clinician–patient education to support equitable uptake. Collectively, this review advances in DCM diagnosis and prognosis, highlighting the role of precision medicine tools for personalized patient care.

## 1. Introduction

Degenerative cervical myelopathy (DCM) is an umbrella term for nontraumatic spinal cord conditions characterized by the progressive compression of the cervical spinal cord (e.g., spondylosis, disc degeneration, ligamentous ossification, spinal canal stenosis) [1]. It is the most common form of nontraumatic spinal cord injury globally, with an estimated prevalence of at least 605 per million and incidence of 41 per million per year in North America [2]. Incidental cervical spinal cord compression is common, with population imaging studies reporting that around 59% of adults aged 40–80 may have had radiological evidence of compression [3]. At the same time, hospitalization rates for cervical spondylotic myelopathy (the most common form of DCM) are ~4.0 per 100,000 person-years and increasing [4]. This is particularly concerning in countries with an aging population, such as Canada and the United States, which may face this healthcare burden disproportionately [2,4]. Ultimately, due to differences in terminology and recognition challenges, the exact incidence of DCM is difficult to determine [3].

The term “degenerative cervical myelopathy” was formally introduced to standardize the diagnosis across various aetiologies of similar clinical presentations [1]. DCM is now recognized as a spectrum of related degenerative disorders that manifest with similar clinical features of spinal impairment and share a similar natural history [5].

Importantly, DCM is generally a progressive condition. Without intervention, many patients experience neurological decline over time, though the rate and course of decline are variable [6]. Classical natural history studies found that approximately 75% of patients deteriorate in a stepwise fashion, characterized by periods of stability separated by episodes of rapid worsening [6]. Only ~20% of patients with DCM exhibit slow, steady progression, and ~5% remain stable long-term after the initial symptoms [7]. Interestingly, up to half of patients managed non-operatively with early DCM showed spontaneous improvement, underscoring the heterogeneity of DCM’s natural history, although this is not well understood [7]. Due to DCM’s unpredictable natural history, particularly in milder forms of the disease process, there is an increasing emphasis on developing practical predictive tools and biomarkers of disease progression. Ultimately, this will enable a precision medicine approach to care, tailoring treatment, timing of surgery, and patient goals to their risk of deterioration. Advancements in clinical scoring, imaging, and machine learning (ML)-based tools are now being leveraged to identify which patients are likely to worsen and therefore merit early intervention, versus those who may be observed safely [8].

The purpose of this review is to synthesize landmark evidence on the clinical presentation, prognostic markers, advanced imaging, and ML tools that have been developed for DCM. While precision medicine is often highlighted as a key area of research and clinical practice within DCM, there have been no reviews to synthesize the current progress in how these tools have impacted DCM management. In developing this understanding, we aim to identify knowledge gaps and key advancements to support DCM management.

## 2. Methodology

This narrative review was conducted by searching PubMed and EMBASE for English-language articles published between January 2010 and October 2025 using combinations of the terms ‘degenerative cervical myelopathy’, ‘spinal canal stenosis’, ‘machine learning’, ‘precision medicine’, and ‘quantitative MRI.’ Reference lists of included articles were hand-searched to identify additional studies. Following manual review, studies were included based on their significance in contributing to the field and their alignment with the review’s objective. Studies were not exhaustively included, but rather were selected to provide a broad, interpretive overview of the evidence. Studies were included if they (a) involved patients with clinically or radiographically diagnosed DCM or pre-myelopathic cervical spinal canal stenosis; (b) reported predictors of disease progression, surgical outcome or neurologic deterioration; (c) described macrostructural or quantitative imaging biomarkers, clinical risk factors, or machine learning models related to DCM outcomes; (d) were original research articles, prospective or retrospective studies, or systematic reviews or narrative reviews that provided primary data. Papers were excluded if they were (a) non-English papers; (b) case reports or case series papers; (c) conference abstracts, editorials, letters, or opinion pieces; (d) animal studies; (e) studies dealing exclusively with traumatic spinal cord injury, thoracic or lumbar stenosis, or paediatric congenital anomalies without DCM.

## 3. Epidemiology and Risk Factors

DCM predominantly affects middle-aged and older adults due to the cumulative degenerative changes that an individual faces across the lifespan. Men are slightly more commonly affected than women in some studies [9], as a result of overuse across the lifespan and higher rates of spondyloarthritis. Socioeconomic factors can influence postoperative recovery and return to work. A Swedish registry study found that older age and physically demanding occupations were associated with delayed return to work after DCM surgery [10]. However, neurological recovery remained favourable across body mass index categories.

While DCM is primarily degenerative/mechanical, studies suggest a genetic component in susceptibility to specific degenerative changes. For example, polymorphism in matrix metalloproteinase-2 (MMP-2) and collagen IX genes has been linked to greater disc degeneration [2], while collagen VI and XI variants have been associated with ossification of the posterior longitudinal ligament (OPLL) [11]. Such genetic factors may predispose an individual to earlier or more severe spinal canal narrowing and myelopathy. Several congenital conditions, like a congenitally narrow spinal canal, Down syndrome, and Klippel-Feil syndrome, increase the risk of developing DCM later in life [2]. It remains unclear whether the relative impact of genetic components outweighs environmental degeneration, although clinical experience suggests a greater environmental role than a genetic one.

There are significant occupational and lifestyle risks to DCM development, in which long-term axial loading of the cervical spine can accelerate degenerative changes. Notably, carrying heavy loads on the head is associated with earlier cervical spondylosis and myelopathy [12]. Continually dynamic stress, such as chronic neck flexion and repetitive neck flexion/extension, has also been shown to add significant stressors to the cervical spinal cord [1,2]. This is exacerbated by high smartphone use, particularly among younger patients who have shown earlier presentations of myelopathic signs as a result of chronic flexion [13]. Other occupations, such as those who chronically engage their upper trunk muscles, are at risk of progressive degeneration [14]. Smoking and poor nutrition have been identified as factors that could exacerbate disc degeneration and worsen recovery, thus indirectly elevating DCM risk. It is also important to note that smoking resists osteoblastic activity in the vertebrae, leading to worse post-surgical outcomes [15] and limiting natural recovery in degenerative spinal conditions [16].

The existence of concurrent spinal pathologies also increases the likelihood of cervical myelopathy, such as in patients with coexisting degenerative lumbar or thoracic ossification, often termed tandem spinal stenosis [17,18]. Systemic conditions, such as diabetes, worsen DCM outcomes due to their impact on the microvascular integrity of the cord [1,2]. In a recent analysis of rapid DCM progression, vascular risk factors (e.g., prior cerebrovascular events) and metabolic conditions such as diabetes and hypertension were enriched in patients who deteriorated rapidly [19]. The interplay of multiple disease processes in DCM outcomes further outlines the importance of a precision medicine approach to treatment and prognosis.

Beyond the well-known risk factors, emerging evidence highlights the role of posterior element degeneration. A recent 2023 study found that facet joint articular irregularity was the strongest independent risk factor for rapidly progressive DCM among patients [19]. Other radiographic factors associated with faster progression included dynamic segmental translation > 3.5 mm, the presence of cervical kyphosis or focal angulation > 4–5 degrees, and multilevel degeneration [20,21]. Ultimately, this suggests that not only do spinal canal dimensions play a crucial role in predicting degeneration, but also the health of the facet joints and overall alignment influence how quickly myelopathy progresses.

## 4. Pathophysiology of DCM

The pathophysiology of DCM is complex and multifaceted, with many contributing pathways to neurologic injury. The primary mechanism of neural injury in DCM is mechanical compression of the cervical spinal cord by degenerative (osteophytes, disc protrusion, hypertrophied ligaments, or OPLL). This form of static compression causes direct deformation of the cord and, when severe, disrupts normal axonal transport and blood flow, leading to ischemia [22]. Compressive forces are more commonly seen at the anterior cord from disc protrusions/osteophytes and posteriorly from hypertrophy or buckling of the ligamentum flavum. In severe and chronic compression, evidence of demyelination, axonal loss, and neuronal death can be seen in the spinal cord [23].

While static compression is more commonly observed, particularly in surgical DCM patients, dynamic factors play an increasingly key role in understanding DCM disease processes. Cervical spine motion (flexion-extension) can exacerbate cord injury through repetitive shear and strain, especially if there is segmental instability. Stretching of the spinal cord over spondylotic ridges during extension and buckling of the ligamentum flavum during flexion, causing transient cord impingement, are two key dynamic injury mechanisms seen in DCM [23]. Ultimately, this repetitive microtrauma contributes to the cumulative neuronal damage experienced by the cord even in the absence of acute injury. These dynamic factors can be exacerbated in patients with abnormal cervical curvature or who may have excessive segmental mobility [24]. This was shown in an animal model, where an induced cervical kyphotic deformity led to significant demyelination and neuronal loss in the cord [23].

Chronic and location-dependent compression can also compress intramedullary microvasculature and/or anterior spinal artery circulation, causing chronic ischemia of the spinal cord [23]. This ischemia has been shown to cause grey matter cell death from reduced perfusion [23]. Spinal cord ischemia can also result in impaired metabolic waste removal, resulting in further tissue injury [22,23]. Chronic cord ischemia in combination with mechanical cord insults creates a progressive degeneration resulting in myelin degeneration, gliosis, and apoptotic cell loss [22].

Concurrent with the primary cord injury pathway described, a cascade of secondary injury mechanisms is thought to occur in DCM. Through the mechanical and ischemic stresses of DCM, a biochemical trigger response, including inflammation and oxidative stress, is activated. Experimental models show an upregulation of inflammatory mediators (e.g., NF-kB pathway components) and matrix metalloproteinases in the compressed cord [22]. In addition to this inflammatory stress, there is evidence that in severe DCM processes, the blood-spinal cord barrier may be disrupted, and infiltration of immune cells may exacerbate tissue damage [22]. In addition to degeneration at the level of the lesion, Wallerian degeneration has been shown to extend myelin loss above the compression in a retrograde manner, indicating upstream effects of a focal compression [25]. In addition to mechanical and ischemic mechanisms, painful degenerative discs exhibit acidic pH and lactate accumulation. Low pH stimulates nuclear pulposus cells to release pro-inflammatory cytokines and neurotrophins, promoting catabolic pathways and pain [26].

Interestingly, the central nervous system attempts to adapt to chronic spinal cord injury through supraspinal neuroplasticity. Patients have been shown to exhibit corticospinal tract reorganization in the motor cortex and brainstem to maintain function despite compromise [27]. Functional MRI studies have revealed compensatory increases in motor cortical activation and preserved alternate pathways in DCM patients [28,29]. Termed “corticospinal reserve capacity,” it may explain why certain individuals remain clinically mild or asymptomatic for long periods despite exhibiting significant cord compression [27]. However, this reserve is not consistently demonstrated and eventually exhausted, at which point rapid clinical deterioration occurs. The variability of each person’s reserve capacity and injury tolerance explains the highly individual nature of DCM progression.

## 5. Clinical Presentation and Diagnosis

DCM often presents insidiously with early symptoms of subtle hand clumsiness (fine motor task difficulty), weakness in the legs, and sensory changes such as numbness or tingling in the hands [3,30]. Patients often report unintentionally dropping objects such as a glass of water or mild coordination trouble long before frank weakness is evident [31]. This is in contrast to many clinical tools that may overlook these early symptoms in favour of gross symptoms, such as gait problems or severe paraesthesia. Neck pain and radicular pain can co-occur if nerve roots are compressed (myeloradicuopathy) [30,31]. In more advanced cases, signs of upper motor neuron dysfunction appear, such as spasticity in the legs and arms, frequent falls, and urinary/bladder issues [32].

In the diagnosis of DCM, key myelopathic signs may help to elucidate the severity of the disease. Classic signs of cervical myelopathy include hyperreflexia, a positive Babinski sign, Hoffman’s sign, and an inverted brachioradialis reflex [30,31,33]. These signs indicate corticospinal tract involvement; however, their absence does not necessarily mean that cervical myelopathy is not present. Importantly, approximately 20% of patients with confirmed cervical myelopathy may lack clear upper motor neuron signs on exam, particularly in mild DCM [2,34,35]. Gait assessment (tandem walking, toe–heel walking) often reveals instability even when leg strength is full.

Magnetic Resonance Imaging (MRI) is the gold standard for the diagnosis of DCM. MRI typically shows degenerative changes such as disc herniation, osteophytes, ligamentum flavum hypertrophy, or OPLL, causing canal stenosis [36]. T2-weighted (T2w) MRI is sensitive for cord pathology and may show intramedullary signal intensity changes at levels of severe compression, indicating edema, inflammation, or gliosis [37]. The absence of a cord signal change does not mean these processes are absent. A T2 hyperintense signal within the cord is a red flag that myelopathic changes are present. In T1-weighted (T1w) images, hypointensity can be seen in severe chronic changes, although more rare, and may indicate irreversible cord damage [36]. Plain radiographs and CT scans can help identify contributory factors, such as instability or ossification, and may be used for surgical planning; however, they do not visualize spinal cord morphology as effectively.

Neurophysiologic studies may be used as supportive diagnostic tools, such as somatosensory evoked potentials (SSEPs) and motor evoked potentials (MEPs), to detect subclinical dysfunction in the dorsal and corticospinal tracts, respectively [38]. Prolongation of evoked potential latencies or dispersed waveforms suggests conduction impairment and has been shown to be a risk factor for future myelopathy in patients with asymptomatic stenosis [38,39]. While isolated pyramidal signs may not be associated with MEPs in cervical myelopathy, their specific role remains to be explored further, particularly in advanced disease modelling, as discussed later [40]. Electromyography (EMG) may demonstrate chronic cervical motor neuron changes, but it is less commonly used clinically, as it is unlikely to alter a patient’s treatment. While neurophysiological studies are generally not routine, they can aid in understanding disease processes in unique patient cases or research settings. These tools may also be used intraoperatively to monitor spinal cord and nerve function during surgery.

Early DCM can be difficult to diagnose because symptoms are mild, nonspecific, and may be attributed to normal “aging”. Patients often present to primary care settings with limited impact on quality of life initially, and it is not uncommon that DCM is misdiagnosed as carpal tunnel syndrome, peripheral neuropathy, or cervical spondylotic radiculopathy [31]. This is further exacerbated by long healthcare wait times and imaging wait times for non-emergent cases. A comprehensive assessment, combining history, physical examination, and imaging, is essential for the early and accurate identification of DCM [31].

## 6. Disease Severity Classification and Natural History

With a wide range of presentations, effective severity grading is essential in the management and triage of DCM. The most widely used grading scale is the modified Japanese Orthopaedic Association (mJOA) score, which is broken into four distinct sections evaluating the motor function in arms and legs, sensory function, and sphincter function [12]. Higher scores indicate milder impairment. This score is stratified into mild DCM (mJOA 15+), moderate DCM (mJOA 12–14), and severe DCM (mJOA < 12) [41]. This severity classification has been widely used in clinical practice guidelines to identify patients recommended for surgery versus those who may opt for watchful waiting [42]. While higher preoperative severity consistently predicts poorer outcomes, new data suggest even mild DCM carries the risk of progression [43]. The Nurick grade is another classic scale that focuses on gait dysfunction as it relates to a person’s capacity for employment, and it is a seven-point scale. Both scales are widely used, although the mJOA has become the clinical standard due to its more comprehensive assessment of a person’s neurological status. Determining baseline severity is important as it has been shown to correlate with postoperative outcomes [2]. There remains clinical uncertainty regarding the management of patients with mild DCM [42].

The natural history of DCM without surgery is notoriously unpredictable, as previously described. On average, classic longitudinal studies such as Clarke and Robinson [44] showed that the majority (70–80%) of patients will worsen neurologically over time if untreated. An important subset of patients includes those with significant cervical cord compression on imaging but no overt symptoms (often termed asymptomatic cord compression). These individuals are at risk of developing DCM in the future, with prospective data suggesting a cumulative risk of approximately 8% at 1 year and 22% at a median follow-up of 44 months [7]. Over a decade, this could translate to one in four such patients becoming myelopathic. Certain risk factors in this patient group can strongly indicate possible deterioration, such as the presence of intramedullary T2 signal change, which may suggest underlying cord stress.

Mild DCM patients present another patient group with clinical management challenges. While mild DCM patients can have periods of stability, studies indicate many will eventually worsen [45,46]. There is no reliable way to identify which specific individuals are at low risk of deterioration compared to those at high risk based solely on clinical examination or T2-weighted imaging. This uncertainty in natural history drives the search for predictive imaging and biomarkers of deterioration in this patient population [43]. Additionally, the risk of acute deterioration in this patient group as a result of minor traumas or falls is increased, leading to the question of safe monitoring. This risk–benefit balance underlies the controversy in managing mild DCM.

Current clinical guidelines recommend surgical decompression for moderate and severe DCM, as the evidence strongly supports better outcomes with surgery in these groups [42]. For mild DCM, guidelines acknowledge a knowledge gap, and optimal management is unclear [42,47]. Recent guidelines have suggested the use of advanced imaging as a means to more closely monitor this patient population [47]. While some clinicians advocate for early surgery to prevent progression, others prefer initial conservative management in mild DCM. An international survey of spine specialists found no consensus on the treatment of mild DCM and asymptomatic cord compression, highlighting an important knowledge gap and research space [48].

## 7. Current Management Challenges and Predictive Tools

Currently, surgical decompression is the only proven effective treatment of DCM [49]. Surgery prevents further damage and often yields substantial neurological improvement, particularly in cases of moderate to severe DCM. Procedures such as anterior cervical discectomy and fusion (ACDF) or posterior laminectomy/laminoplasty remove the offending pathology, decompress the cord, and stabilize the spine. Standard ACDF is typically completed under general anaesthesia in roughly two hours with minimal blood loss and tissue disruption. Non-operative measures (collars, physiotherapy, etc.) do not reliably prevent deterioration and are often mainly used for symptomatic relief [50]. That being said, surgery carries with it risks and a significant recovery burden, making it challenging to balance the risks and benefits in mild DCM patients. The key clinical challenge lies in mild DCM and patients with pre-myelopathic stenosis who currently exhibit minimal impairment but an uncertain future. Compounding this challenge is the impact on quality of life and work disability. DCM is a leading cause of work disability, and early intervention may yield long-term benefits to individual finances [51]. A recent cost–utility analysis in Canada found that performing early surgery in mild DCM was cost-effective in the long run, due to prevention of quality-of-life loss and disability [52]. Early intervention yielded more quality-adjusted life years on average, despite the upfront surgical costs [52,53]. However, this analysis assumed an average risk of deterioration. If patients at higher risk could be identified, it could further justify early intervention while sparing surgical intervention for those at lower risk.

**Figure 1:**
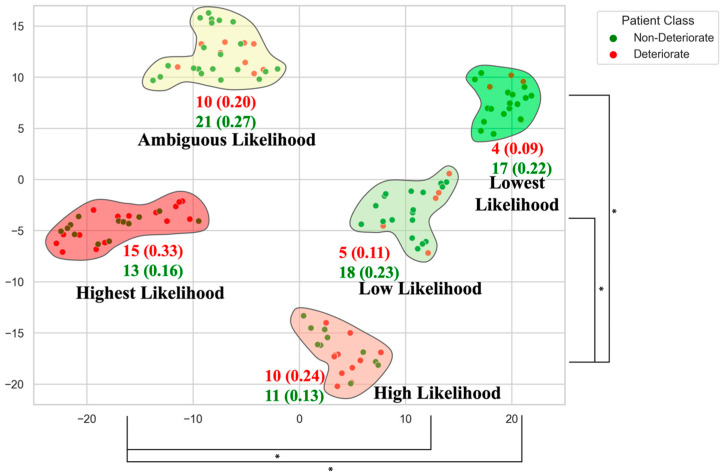
Clusters of non-operative mild DCM patients stratified by relative risk of neurological deterioration. Each cluster’s deterioration likelihood ratio is shown with the number of patients deteriorating in that group. This data-driven phenotyping illustrates how certain patient subgroups have higher deterioration risk, informing tailored surveillance and possible early intervention. (*; *p* < 0.05) [55].

In response to these individualized patient risk challenges, researchers are developing a variety of predictive approaches involving clinical risk scores, advancing imaging metrics, and predictive ML modelling. The goal of these predictive models is to support clinical decision-making by providing objective markers of deterioration that provide an individualized risk assessment [54]. Additionally, predictive models have been able to map DCM patient phenotypes at greater risk of deterioration (Figure 1). This follows a precision medicine approach of individualized care and may yield improved outcomes over general guidelines.

## 8. Conventional Prognostic Factors

A longstanding and strong predictor of outcomes in DCM is baseline severity of cervical myelopathy. Interestingly, patients with a higher mJOA score preoperatively tend to see greater postoperative improvement, likely due to less permanent cord damage [2]. A systematic review of 91 studies identified preoperative severity and longer symptom duration as the most consistently reported predictors of poorer outcomes [16]. Prolonged duration of symptoms before surgery has been linked to worse neurological outcomes, likely due to irreversible cord changes and spinal cord atrophy [30]. Studies have shown that symptom duration >12–18 months, patients recover less than those treated earlier; however, the exact threshold varies [2].

Advanced age has been shown to be a negative prognostic factor for recovery after surgery due to decreased neuroplasticity and increased comorbid conditions [2]. Furthermore, smoking has been cited as a factor correlating with worse surgical outcomes [56]. As previously highlighted, T2 hyperintensity and T1 hypointensity have been linked with worse prognostic outcomes in patients [37].

**Figure 2:**
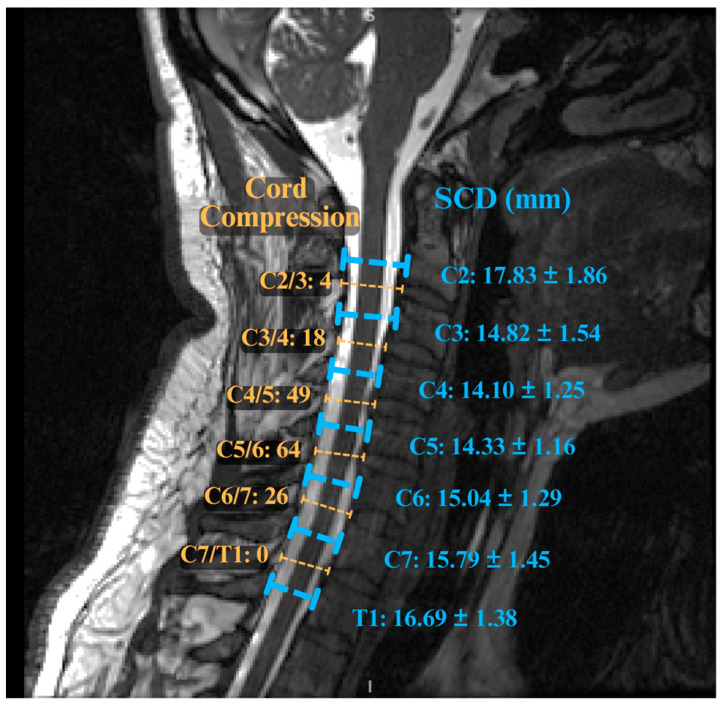
Mean spinal canal diameter (mm) at each cervical vertebral level in a cohort of DCM patients. This illustrates congenital differences in canal size and the relative distribution of compression at each cervical level [55].

Several radiological metrics have also been shown to have prognostic value. A narrow spinal canal diameter or a small cord cross-sectional area has been linked with worse outcomes [37,55]. Variable spinal canal sizes at different vertebral levels also add to this complexity (Figure 2). Multilevel cord compression (>2 levels) is also associated with a greater risk of postoperative non-improvement [55,57,58]. In one multivariate analysis, the degree of C2–C7 kyphosis was an independent predictor of failure to improve after surgery, with each 10-degree increase in kyphosis associated with a higher risk [59].

Several clinical signs have also been shown to correlate with worse prognostic outcomes, such as severe gait ataxia [60] and sphincter dysfunction [61]. It is important to note that while many factors have been studied, results across studies have shown great inconsistency [16,61]. The study of comorbid conditions is limited, although some reports find diabetes to predict worse outcomes [62]. Some consider the Nurick scale to be highly predictive, while others do not [16]. This may be as a result of traditional statistical limitations and an inability to consider non-linear interactions. This further underscores the need for more nuanced and multifactorial modelling of DCM predictors.

## 9. Advanced Imaging Markers in DCM

While routine conventional MRI (T1w/T2w sequences) are invaluable for DCM diagnosis and surgical planning, they present great limitations in prognostication. The presence of cord compression and T2 signal change qualitatively indicates myelopathy, but conventional MRI does not provide quantitative microstructural information on spinal cord integrity. Advanced quantitative MRI (qMRI) methods overcome this limitation by providing information on both spinal cord axonal integrity and myelination, thereby better predicting outcomes [63].

Diffusion tensor imaging (DTI) is the most common form of qMRI method used in the DCM literature and provides a measure of the diffusion of water in tissue [64]. This, in turn, can be extrapolated to infer axonal integrity using metrics such as fractional anisotropy (FA) and mean diffusivity (MD). In the spinal cord, intact white matter tracts restrict water diffusion anisotropically. Therefore, measuring a decrease in FA at the compression level would suggest disruption of the axonal architecture. Studies have shown that lower FA in DCM patients correlates with worse neurological function [25,64]. Ellingson et al. [64] reported that DTI parameters predicted functional impairment even in mild to moderate cervical spondylotic myelopathy, indicating its possible use in milder cases. Understanding the degree of microstructural damage provides clinicians with valuable information not visible on routine MRI. Furthermore, early evidence suggests that patients with abnormal DTI findings have poorer recovery, further indicating its potential as an imaging prognostic marker [65].

**Figure 3:**
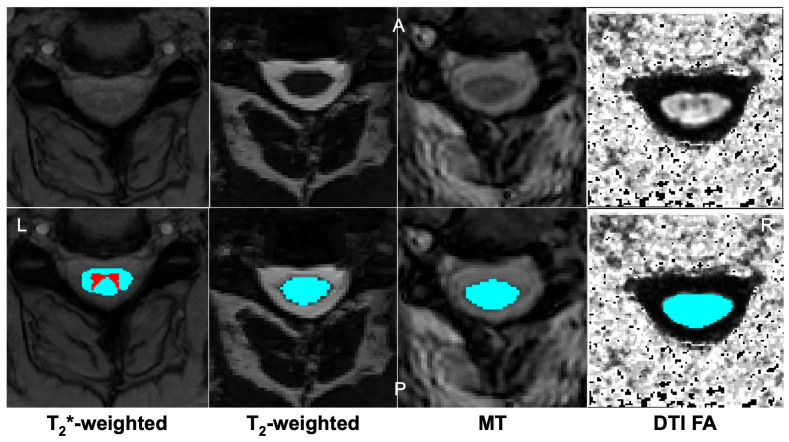
MRI scan visualization and automated segmentation using the Spinal Cord Toolbox. Images were captured at the C3 level from a representative control patient. The top thumbnails show the image without segmentation, and the bottom images include segmentation. (**Left** to **Right**) T_2_*-weighted grey matter (red) and white matter (blue) scan, T_2_-weighted, magnetization transfer scan, and fractional anisotropy [69]. MT = magnetization transfer, DTI = diffusion tensor imaging, L = left, R = right, A = anterior, P = posterior.

Magnetization transfer (MT) MRI examines the exchange of magnetization between free water and macromolecule-bound water. Through this examination, a myelin content surrogate can be derived, indicating the degree of myelination in the spinal cord. Magnetization transfer ratio (MTR) is the key quantitative metric derived and is reduced in demyelinated tissue [66]. In DCM, regions of chronically compressed cord have been shown to have lower MTR and myelin content [67]. MTR has also been shown to be decreased in mild DCM patients before changes in DTI can be detected, indicating its utility in early deterioration detection [43]. A 2025 systematic review highlighted that MT imaging, along with DTI, was an emerging tool to improve DCM assessment [68]. More recent work has shown that the degree of MTR reduction within dorsal columns has been linked to the severity of proprioceptive deficits [43]. Figure 3 demonstrates axial scans of these advanced imaging scans and post-segmentation processing.

Other qMRI techniques have been used, such as myelin water imaging (MWI) to directly quantify myelin content and MR spectroscopy (MRS) to measure metabolites. Although these tools have primarily been used within an academic context, they offer further insights into understanding the role of microstructural components in DCM deterioration [70]. Initial studies using MWI have shown correlations between lower myelin water content and worse DCM impairment [70]. Functional MRI has also been utilized in the context of DCM, demonstrating reorganization in the brainstem and cortex. A 2020 study showed increased brainstem connectivity in DCM patients [28].

Another longstanding imaging marker of deterioration has been spinal cord cross-sectional area (CSA), typically measured at the C2–C3 level. Cord atrophy is a marker of chronic degeneration, and DCM patients are at greater risk of having reduced CSA compared to normals [63]. Automated analysis tools, such as the Spinal Cord Toolbox [71], enable the automated measurement of CSA in the spinal cord. While these tools have limitations, particularly in sick populations like DCM that exhibit cord compression, they provide cost-effective solutions to otherwise complex-to-compute cord metrics.

Despite their promise, qMRI techniques like DTI and MT imaging are not routine in clinical practice for DCM. They require longer scan times, specialized pulse sequences, and advanced analysis that is not currently widely available. Ongoing efforts to standardize scanning protocols are underway [72] to facilitate the development of large, high-quality datasets. As these techniques become more accessible, they hold potential to significantly refine prognostication by directly measuring the spinal cord’s tissue integrity and highlighting microstructural damage. Notably, the greatest prognostic value may emerge when these advanced imaging markers are combined with clinical variables in multimodal predictive models. Table 1 provides a comparative summary of key advanced imaging modalities used in DCM.

## 10. Machine Learning and Predictive Models in DCM

A recent increase in artificial intelligence (AI) and ML tools being used in the academic literature has led researchers to question their utility in a DCM context. Given the multifactorial nature of DCM, ML approaches have been increasingly applied to identify complex patterns and improve predictive accuracy. ML tools provide the added capability of analyzing high-dimensional combinations of clinical, imaging, and demographic features. The ultimate goal of many studies has been to develop a model capable of predicting postoperative outcomes at an individual level in patients with DCM. A 2019 systematic review found that of all ML studies in spinal cord injury, only six focused on nontraumatic spinal cord injury, indicating its novelty [73]. Furthermore, most ML applications have focused on predictive postoperative outcomes, with few addressing the current clinical limitations in better predicting the natural history for patients with mild DCM [73].

In 2021, a study published in the Spine Journal analyzed 193 mild DCM patients utilizing ML approaches to predict postoperative health-related quality of life improvements [74]. The best model achieved an AUC of 0.78 for predicting changes in SF-36 quality of life [74]. Notably, this model only included clinical variables. In a complementary study, an ML model was trained to identify patients’ risk of worse functional status after surgery [75]. This model also used preoperative clinical factors and found that long symptom duration and severe baseline disability were two key risk factors [75]. These studies provided two early ML approaches used in the prediction of DCM outcomes.

More recent efforts have aimed to incorporate both clinical and imaging features into predictive models. A 2022 single-center study used multivariate modelling on 183 surgical patients, considering a wide array of preoperative clinical and MRI factors [76]. They found a combination of imaging parameters, such as the number of levels with cord compression, the presence of cervical kyphosis, and the length of intramedullary T2 lesion, together provided the best predictors of failure to improve after surgery (AUC 0.82) [76]. While this model incorporated imaging findings, it was limited to radiographic findings and did not incorporate advanced imaging findings into the model.

As previously highlighted, a critical under-addressed question surrounds the management of mild DCM patients and their deterioration status if left untreated. A novel 2025 study looking at a cohort of non-operative mild DCM patients implemented a supervised ML approach utilizing both clinical and advanced imaging metrics to predict which patients would be most at risk of deterioration [43]. Through a combination of clinical, DTI, and MT imaging, the best predictive model had a balanced accuracy of 83% (AUC 0.87) [43]. While this study showed promising results in a mild DCM context, the limited patient cohort of 43 patients reduces the model’s generalizability and requires a multi-centre research effort.

Precision medicine and modern ML in healthcare emphasize not just the capacity for models to predict but their inherent interpretability and capacity to explain how the model made its prediction. While model explainability tools like SHAP (Shapley Additive Explanations) and LIME (Local Interpretable Model-Agnostic Explanations) exist, they are not often used in DCM research studies, leading to increased clinical skepticism. Some classical models (e.g., random forests) have built-in interpretability via feature importance rankings, and indeed, researchers have leveraged this. For instance, in one classification of surgical decision-making, a feature importance analysis showed that the ML algorithm’s top predictors were the same factors surgeons typically consider [77]. The use of external explainability tools is an essential component of future research studies and ML models, with the hope of clinical implementation.

In another study, a LightGBM model achieved an AUC of 0.74 for predicting clinically meaningful recovery after decompression, hinting at the utility of more advanced ML models over traditional logistic regression models [78]. In addition to predicting postoperative outcomes, ML models have been applied to classify clinical-level treatment decisions (conservative vs. anterior vs. posterior surgery) with high fidelity (multiclass AUC of 0.90) [77]. Through a feature importance analysis, it was shown that the model utilized similar clinical tools in making its decision [77].

While ML models can be used as predictive tools, they may be able to expedite spinal cord analysis pipelines. Deep learning tools, such as DeepSeg [79], are used to automatically segment the spinal cord and detect spinal canal stenosis, providing in-depth morphological metrics and highlighting areas of signal change [71]. These tools may provide a support role in patient triage, highlighting patients who may be at greater risk of deterioration.

Table 2 summarizes key ML studies in DCM prognostication, highlighting their sample characteristics, input features, outcome targets, and performance metrics.

### Current Model Limitations

Importantly, most predictive models are trained on retrospective single-center datasets and demonstrate moderate performance (AUC ~0.7–0.8). This leaves a large gap for model generalizability and validation, raising concerns about model overfitting. Furthermore, as previously described, the inherent “black box” nature of many deep learning methods limits clinical trust and model interpretability [73,75]. In addition to these limitations, there exist limitations in dataset quality, with many centers having different collection protocols, scanning methodology, and clinical metric collection. This data heterogeneity results in incomplete datasets and significantly impedes ML model training and validation. Harmonizing data elements across studies is therefore critical.

## 11. Discussion

While many reviews of DCM have explored disease pathophysiology, progression, and clinical findings, there have been no reviews to synthesize these areas of research within a critical lens of precision medicine and ML. As ML and personalized patient care models rapidly develop, this review serves as an integrated perspective providing both clinicians and bioinformaticians with an overview of the current DCM precision medicine landscape.

### 11.1. Unmet Clinical Needs

Despite advances, there remain critical unmet needs in DCM care. Studies have noted that DCM diagnosis is frequently missed or delayed in general practice due to its subtle and nonspecific presentations [31]. This underlines the importance of more sophisticated and individualized screening tools, particularly in a patient population with minimal symptoms but who may be at risk of neurological deterioration. Additionally, despite advances, there is a lack of neuroprotective treatments for DCM. Surgery prevents further cord compression, but pre-existing damage, particularly axonal damage, often remains unaddressed. The failure of past trials, such as riluzole, partly stemmed from limitations in outcome measures, and further exploration into adjuvant therapies may be warranted [80].

A practical issue in the management of DCM involves patient follow-up, particularly in patients who are managed through watchful waiting. Currently, there exists no consensus on surveillance frequency or modality, making the modelling of DCM’s natural history difficult and follow-up guidelines unclear [57]. Without standardized follow-up protocols and sensitive detection of deterioration risk, patients may continue to undergo conservative management despite the need for surgical intervention. Predictive models could help inform these decisions. For example, a patient identified by a model as high-risk for deterioration could be monitored more closely, whereas a low-risk patient might safely undergo less frequent follow-up. ML may play an essential role in addressing this need through multivariate modelling that provides an individualized risk estimate, particularly for mild DCM patients, thereby guiding the timing of intervention in an evidence-based manner.

### 11.2. Controversy in the Timing of Mild DCM Intervention

Perhaps the most prominent controversy in DCM management revolves around patients with mild myelopathy. The clinical course in this group is remarkably heterogeneous, with some mild DCM patients remaining neurologically stable and others showing marked deterioration within months to years [2]. This variability fuels uncertainty surrounding the question of early surgical intervention in this patient population, as evidenced by a lack of consensus within the surgical community [48]. While mild cord compression may not demonstrate axonal damage, there is evidence of possible early demyelination [70], and the cord’s capacity to remain with existing damage is unclear [25]. That being said, proponents of initial conservative management note that surgery carries risks, and not all mild patients progress quickly. Kadanka’s classic trial suggests no short-term difference in outcomes, even demonstrating some patients stabilize with non-operative care [46]. That being said, this uncertainty partially stems from the fact that clinicians cannot currently stratify which mild DCM patients are more at risk of deterioration based on clinical signs and T2w MRI images alone [36]. If more sophisticated imaging and predictive tools were available, this could help guide informed practice.

### 11.3. Barriers to the Development of Novel Tools

While our review is optimistic about the role of ML models in identifying patients at risk of deterioration, there lie challenges in translating these tools into everyday practice. A primary challenge exists around the issues of validation and evidence. Clinicians rightfully demand high levels of evidence and validation before changing practice, which cannot currently be met due to dataset limitations and a lack of multi-centre studies. Advanced diagnostics often require specialized equipment and technical support, both of which are limited within healthcare systems. DTI and other qMRI sequences may not be readily available outside of major academic centers or in community hospitals, and access to care becomes a significant concern in equitable risk stratification. Furthermore, the introduction of new models, algorithms, and tools that may disrupt traditional workflows or require clinician time may be met with resistance. To mitigate these concerns, tools must be cost- and time-effective while remaining as explainable decision support rather than directives. Encouragingly, when ML models are understandable and aligned with clinical reasoning, surgeons have shown willingness to consider them [77].

Most current models have been developed on relatively narrow datasets. Multi-centre collaboration is needed to overcome barriers of generalizability and data harmonization. Precision medicine in DCM will also require a better data infrastructure, allowing centers to combine imaging, laboratory, and clinical measurement data. Efforts to build DCM registries are challenging but have begun, such as the AO Spine RECODE initiative and the CSORN initiative [81]. Such registry initiatives aim to improve generalizability by ensuring that predictive models are trained and validated on standardized, representative datasets.

### 11.4. Evolution in DCM Management

DCM management is evolving from a reactive approach of treatment to a proactive, precision-based approach. While it is still common to wait for significant neurological decline before intervention, new initiatives aim to predict and prevent decline by treating the right patient at the right time. An area of emerging consensus is the need for improved classification in DCM, with new tools that can more comprehensively capture a patient’s disease status, risk of deterioration, and impact on quality of life.

Another point of synthesis in the literature surrounds the importance of patient-centred outcomes. There is an impetus to incorporate high-fidelity functional measures, specific clinical tools, and high-quality patient-reported outcomes. As highlighted, traditional measures such as the mJOA currently have many limitations in accurately capturing a patient’s disease status in a comprehensive manner [82]. Measures that more comprehensively assess hand dexterity, coordination, and overall functional status, such as the Purdue Pegboard Test [83], the triangle step test [84], and the Minnesota Manual Dexterity Test [85], are all emerging tools that help to characterize the degree of functional deterioration that a patient may face.

**Figure 4:**
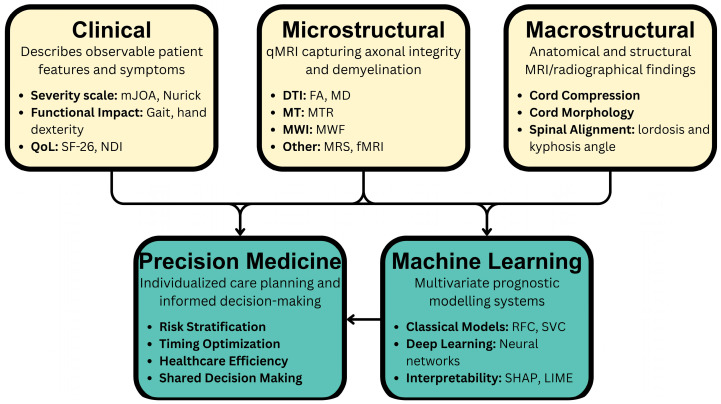
Integrated multimodal precision framework incorporating phenotypical clinical metrics, macrostructural metrics, and microstructural metrics.

Importantly, the integration of clinical, imaging, and computational predictors, as highlighted in this review, allows for a new framework for DCM care. By synthesizing information across these domains, clinicians can stratify risk more accurately than with individual metrics. Broadly, categories of metrics can be considered as clinical phenotypes (symptoms and exam findings), macrostructural (e.g., cord compression length, number of compressed levels), and microstructural (e.g., FA or MTR values). Figure 4 illustrates this integrated multimodal approach, showing how clinical, macrostructural, and microstructural inputs are aggregated by ML models to generate personalized risk predictions and outcome measures. While these tools are currently in pre-clinical implementation, practically, they could identify DCM patients at high risk of deterioration, leading to closer surveillance or more timely intervention.

### 11.5. Limitations

As a narrative review, our article may be subject to selection bias as no formal evidence grading was applied. In the nature of the narrative review, we did not follow PRISMA guidelines and did not exhaustively include eligible studies. We focused on salient contributions, and therefore, some relevant studies may not be discussed. Moreover, the field is evolving rapidly; thus, conclusions drawn now will require updating as new evidence emerges. Finally, because many advanced precision medicine tools still have limited data, prognostic frameworks highlighted in this review are primarily conceptual and based on early data. Many of these tools require multi-centre validation and larger cohorts to be implemented clinically.

## 12. Future Direction

On the horizon of DCM are several promising technologies poised to further improve DCM management. Ultra-high field and functional imaging tools offer high-fidelity scans of cord microstructure and connectivity, detecting early myelopathic changes before clinical onset. The integration of wearable technology enables the dynamic assessment of spinal cord morphology, providing another key yet underreported avenue for assessing spinal cord damage. Additionally, the use of advanced neurophysiological assessments offers another complementary tool for direct functional measures of corticospinal tract integrity. Lastly, the use of artificial intelligence in imaging and clinical support is a growing field in which ML models may be implemented within clinical practice to aid in patient triage, deterioration risk evaluation, and informed surgical decision-making.

The validation of precision medicine tools through clinical studies is the most important next step in bringing these tools to clinical practice. Prospective, multi-center studies must be completed using these tools with continual refinement and adjustment to specific patient populations and their respective unique risk factors. Data sharing efforts nationally and internationally through a collective patient registry will aid in the development of comprehensive tools, identification of robust risk factors, and expedite the validation of clinical tools. Lastly, clinician and patient education on precision medicine tools is essential to ensure clinical uptake and appropriate implementation of these tools.

## 13. Conclusions

Degenerative cervical myelopathy is a complex nontraumatic spinal cord pathology that has presented management challenges, garnering significant academic and research interest over the past 20 years. DCM is entering an era of precision medicine. Traditional prognostic factors like clinical severity and duration are being augmented by qMRI markers and ML models to better predict outcomes on an individual level. This review has highlighted these precision medicine advancements and their collective effort to create a future in which DCM is managed through data-driven and patient-centred approaches. Traditional paradigms of “watch and wait” and “operate now” are continually being refined by tools that help to determine when each decision is appropriate, the time it is appropriate, and for whom it may be appropriate. As we advance, multi-centre validation of predictive models and education on these innovations will be crucial to translate research into practice.

## Figures and Tables

**Table 1:** Comparison of advanced MRI techniques commonly used in DCM.

Imaging Modality	Acquisition Considerations	Key Biomarkers	Findings/Utility in DCM
Diffusion Tensor Imaging (DTI)	Echo planar imaging sequence; moderate scan time (5–10 min); prone to motion and distortion artefacts.	Fractional anisotropy (FA), mean diffusivity (MD), etc., reflecting white matter axonal integrity and water diffusion.	Lower FA and higher MD at the compression level correlate to greater neurological impairment [64]. Low FA has been associated with poorer postoperative improvement [65]
Magnetization Transfer (MT)	Specialized pulse sequence with MT saturation; ~5 min scan time; requires off-resonance pulse calibration.	Magnetization transfer Ratio (MTR), indicating myelin and macromolecular content.	Compressed cord regions show reduced MTR, indicating demyelination [66]. MTR changes can appear in mild DCM before DTI changes [70].
Myelin Water Imaging (MWI)	Multi-echo MRI; long acquisition (>10–15 min); high technical expertise needed.	Myelin water fraction relates to the proportion of water trapped between myelin layers.	Studies report lower myelin water fraction in patients with DCM [70], consistent with demyelination. Limited data for this imaging type.

**Table 2:** Key recent machine learning studies for outcome prediction in DCM. These representative studies illustrate the progression from using purely clinical predictors to incorporating imaging features of disease progression.

Study (Year)	Sample (*n*, Population)	Main Predictors	Outcome Predicted	Performance (AUC, Best Model)
Merali et al. (2019) [75]	*n* ≈ 600 (multi-center surgical DCM cohort; data from AOSpine CSM trials)	Clinical and demographic features	Improvement in health-related quality of life (SF-36) after surgery	~0.70 (Random Forest model)
Khan et al. (2021) [74]	*n* = 193 (mild DCM patients, AOSpine CSM trials)	Clinical only	Clinically meaningful improvement in SF-36 score 1-year post-surgery	0.78 (ensemble classifier)
Toop et al. (2023) [76]	*n* = 183 (mixed severity, single center)	Clinical + conventional MRI metrics	Failure to improve neurologically after surgery	0.82 (Logistic regression model)
Zhou et al. (2025) [78]	*n* = 672 (mixed severity, single-center retrospective)	Clinical + MRI signal change	Short-term postoperative outcomes	0.745 (LightGBM model)
Park et al. (2022) [77]	*n* = 304 (mixed severity, single-center retrospective)	Clinical + radiologic metrics	Classification of treatment decision (conservative vs. surgery)	0.92 (Gradient Boosting Model)
Al-Shawwa et al. (2024) [69]	*n* = 524 (mixed severity, single-center retrospective)	Conventional and qMRI metrics	Prediction of disease severity class	AUC not reported. 0.418 balanced accuracy (conventional MRI) 0.733 balanced accuracy (advanced MRI)

## Abbreviations

The following abbreviations are used in this manuscript:

AI	Artificial Intelligence
AUC	Area Under the Curve
CSA	Cross-Sectional Area
CSORN	Canadian Spine Outcomes and Research Network
CT	Computed Tomography
DCM	Degenerative Cervical Myelopathy
DTI	Diffusion Tensor Imaging
EMG	Electromyography
FA	Fractional Anisotropy
MD	Mean Diffusivity
MEPs	Motor Evoked Potentials
mJOA	Modified Japanese Orthopaedic Association
ML	Machine Learning
MRI	Magnetic Resonance Imaging
MT	Magnetization Transfer
MTR	Magnetization Transfer Ratio
MWI	Myelin Water Imaging
OPLL	Ossification of the Posterior Longitudinal Ligament
qMRI	Quantitative MRI
SF-36	36-Item Short Form Health Survey
SSEPs	Somatosensory Evoked Potentials
T1w	T1-Weighted
T2w	T2-Weighted
